# Preparation of BN Nanoparticle with High Sintering Activity and Its Formation Mechanism

**DOI:** 10.3390/molecules29153458

**Published:** 2024-07-24

**Authors:** Qun Li, Kuo Zhang, Xiangming Che, Tengchao Gao, Shuhuan Wang, Guolong Ni

**Affiliations:** 1 College of Metallurgy and Energy, North China University of Science and Technology, Tangshan 063210, China; liq@ncst.edu.cn (Q.L.); z2540512276@outlook.com (K.Z.); 15256510486@163.com (X.C.); 13785052806@163.com (T.G.); wshh88@ncst.edu.cn (S.W.); 2 Tangshan Key Laboratory of Special Metallurgy and Material Manufacture, Tangshan 063210, China

**Keywords:** precursor treatment, h-BN nanoparticles, controllable synthesis, sintering activity, formation mechanism

## Abstract

Hexagonal boron nitride (h-BN) nanoparticles have attracted increasing attention due to their unique structure and properties. However, it is difficult to synthesize h-BN nanoparticles with uniform spherical morphology due to their crystal characteristic. The morphology control by tuning their precursor synthesis is a promising and effective strategy to solve this problem. Especially, the treatment temperature of precursors plays an important role in the morphology and surface area of h-BN nanoparticles. Herein, h-BN nanoparticles with different morphologies were synthesized via regulating the treatment temperature of precursors. The result shows that treatment temperature will affect the microstructure and state of precursor and further influence the morphology of h-BN products. Benefiting from the unique structure, the h-BN obtained using 250 °C precursors shows higher specific surface area (61.1 m^2^ g^−1^) than that of 85 °C (36.5 m^2^ g^−1^) and 145 °C (27.9 m^2^ g^−1^). h-BN products obtained using 250 °C precursors show higher specific surface area than that of 85 °C and 145 °C. The optimal condition for obtaining high-quality spherical h-BN is the pretreatment temperature of 250 °C and sintering temperature of 1300 °C. Importantly, compared with commercial h-BN nanoparticles, the synthesized h-BN nanoparticles show more uniform structure and larger specific surface area, indicating that sintering activity will be greatly improved. Furthermore, the reaction pathway and formation mechanism of h-BN was revealed by DFT calculations. The result shows that the five stationary states and five transition states exist in the reaction pathway, and the energy barrier can be overcome at high temperatures to form a ring h-BN. In view of its simplicity and efficiency, this work is promising for designing and guiding the synthesis of h-BN nanoparticles with uniform morphology.

## 1. Introduction

Hexagonal boron nitride (h-BN) nanomaterials, as a structural analogy with graphite, have attracted considerable attention by virtue of their wide band gap, large surface area, high thermal conductivity and stability, excellent oxidation resistance and good chemical inertness. These unique properties render them widely applicable in ceramics, thermal management, sensing, adsorption and catalysis [1,2,3]. Especially in recent years, with the increasing application demand, great efforts have been devoted to developing novel boron nitride materials with controllable morphology and structure, such as nanoparticles [4], nanonets [5], nanocarpets [6], hollow spheres [7], monoliths [8], whiskers [9] and so on. Among them, spherical morphology, as an important member of the microstructure, plays an irreplaceable role in promoting the development of h-BN materials [10].

To our knowledge, h-BN easily forms a large-size sheet structure during the synthesis process due to its crystal characteristic [11]. Thus, many research works on sheet structure have been reported, and commercial boron nitride also possesses a flake-like structure or fish scale with large aspect ratio at present [12]. Furthermore, the size and morphology of commercially available h-BN nanoparticles are usually non-uniform. These above phenomena make them extremely easy to generate pores and form a triangle supporting structure during the sintering process, which also limits the further improvement of h-BN ceramic properties [13,14]. Research studies show that the size and morphology of h-BN particles have important effects on sintering properties [11]. Zhai et al. reported that grain size is the key factor to determine the fracture strength of ceramics, and the fracture strength decreases with the increase in grain size [15]. In addition, the thermal conductivity of h-BN ceramics is closely related to its microstructure, especially the grain orientation [12]. The spherical h-BN powder is isotropic, which can ensure uniform thermal conductivity in all directions. Thus, it can be inferred that spherical h-BN nanoparticle with uniform and tunable size and morphology may be a promising candidate to solve the above problems due to the unique physical and chemical properties given by morphology and dimension effect [16,17,18]. However, there are still some challenges and technical difficulties in its large-scale preparation, and further exploration and optimization of preparation methods are highly urgent.

From the aforementioned h-BN materials with novel structure, it can be found that treatment conditions and variety of precursors have an extremely important influence on the morphology, microstructure and properties of h-BN nanoparticles. However, studies about the temperature effect of precursors on h-BN product are rare, and the formation process of h-BN from raw materials is undefined and still needs to be further explored. In this work, the h-BN nanoparticles with different morphology and structure were synthesized by regulating the treatment temperature of precursors, and the formation mechanism was presented and discussed. Furthermore, compared with commercial h-BN nanoparticles, the synthesized h-BN nanoparticles show more uniform structure and larger specific surface area, indicating that sintering activity will be greatly improved.

## 2. Results and Discussion

### 2.1. Characterization of Precursors

**Figure 1:**
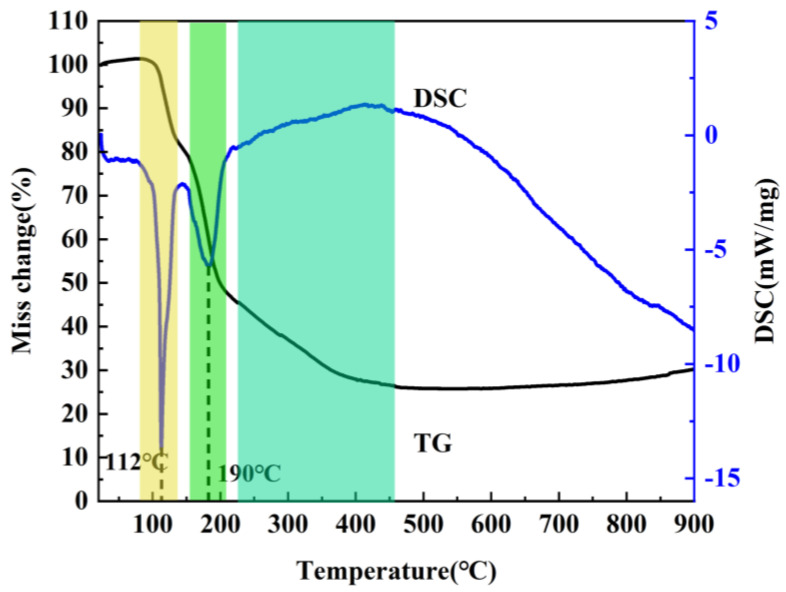
TG–DSC curve of the mixture of H_3_BO_3_ and CO(NH_2_)_2_ under air atmosphere.

It is well known that the precursor has a significant influence on the resultant h-BN. In order to optimize the precursor treatment conditions, the effect of temperature on precursors is discussed. The treatment temperature of precursors is firstly determined by the thermogravimetric analysis of the mixture of H_3_BO_3_ and CO(NH_2_)_2_ at a heating rate of 5 °C min^−1^ under air atmosphere from room temperature to 900 °C. As shown in Figure 1, the whole reaction process mainly includes three weightlessness stages. The first one is at 100–140 °C, and the endothermic peak is mainly assigned to the melting and decomposition of urea. During the process, when the temperature exceeds the urea melting point (132.7 °C), urea immediately produces a large amount of ammonia gas, isocyanic acid and biuret [19], etc., which leads to the rapid decrease of the mass. Meanwhile, the dehydration of H_3_BO_3_ to metaboric acid (HBO_2_) also contributes to the mass decrease. The second one is at 155–200 °C and the endothermic peak corresponding to the decomposition of biuret and residual urea. The third one is at 220–400 °C, and there exist weak exothermic reactions during the stage, which is caused by the further decomposition of HBO_2_ [20,21]. Based on the above results, it can be found that no reaction happens when the temperature is below 100 °C. The transition stage of the first and second stages is 140–155 °C, and the second stage is completely over when the temperature is above 200 °C; in addition, because NH_3_ is in favor of the growth of h-BN along the (002) plane during the synthesis process, and most of NH_3_ has escaped at 145 °C. Also, H_3_BO_3_ and CO(NH_2_)_2_ have decomposed to form a complex intermediate at 250 °C. Therefore, 85 °C, 145 °C and 250 °C were chosen to study the effects of precursor treatment temperatures (PT) on h-BN products.

**Figure 2:**
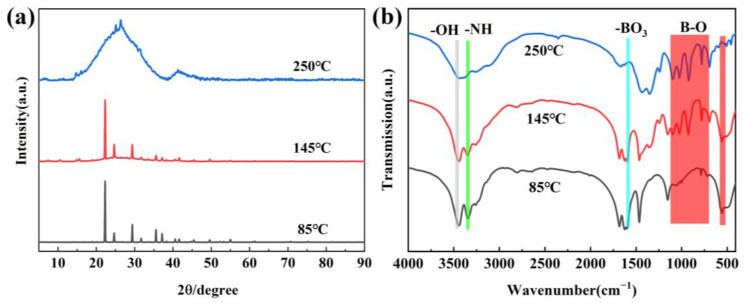
XRD patterns (**a**) and FTIR spectra (**b**) of precursors obtained at different treatment temperatures.

Figure 2a shows the XRD of precursors obtained at different temperatures. When the treatment temperature is 85 °C, the diffraction peaks correspond to H_3_BO_3_ (JCPDS 73-2158) and CO(NH_2_)_2_ (JCPDS 83-136). This is because the temperature is too low for the reaction to occur. With the temperature increasing, the new diffraction peaks of metaborate, biuret and isocyanic acid appear, indicating that the decomposition of H_3_BO_3_ and CO(NH_2_)_2_ has happened, accompanied by a release of CO_2_, H_2_O and NH_3_ gases. When the temperature continuously increases to 250 °C, the peaks of H_3_BO_3_, CO(NH_2_)_2_ and their by-product cannot be found, and two board peaks at 20–30 and 40–45° appear, which reflects that the precursor is amorphous and the new substance has formed. The new substances are composed of the by-product H_3_BO_3_ and CO(NH_2_)_2_, and they can be identified to the boron oxynitride-related one [10]. The result is in accordance with thermogravimetric analysis and further confirms that the temperature chosen is reasonable. The FTIR spectra of the obtained precursors are shown in Figure 2b. By comparison, it can be found that the peaks at 786–1356 cm^−1^ corresponding to B–O/B–OH are very weak at 85 °C [22], indicating that the decomposition of H_3_BO_3_ does not occur at 85 °C and has happened at 145 °C. In addition, two board peaks at 3442 and 3207 cm^−1^ are attributed to –OH and –NH_2_ groups, respectively [23,24]. It is worth noting that the intensity of precursors obtained at 250 °C decreases, and the peaks at 1650 cm^−1^ and 786 cm^−1^ disappear, which further confirms the decomposition of H_3_BO_3_ and CO(NH_2_)_2_ to form new substance [25,26].

**Figure 3:**
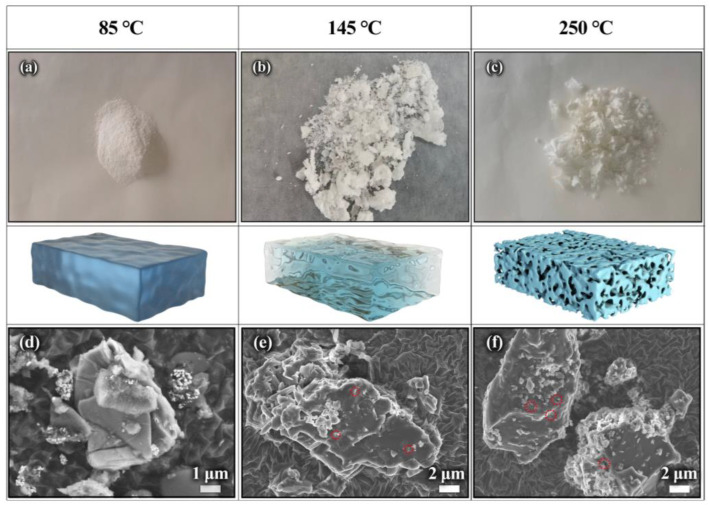
Photographs (**a**–**c**) and SEM images (**d**–**f**) of precursors obtained at different temperatures.

Furthermore, the above phenomenon can also be explained by the precursor photographs in Figure 3a–c. As observed in Figure 3a, the precursor at 85 °C has no obvious change and still keeps the powder state of raw materials. However, the powder transforms into a smooth molten state on account of the decomposition and melting of H_3_BO_3_ and CO(NH_2_)_2_ at 145 °C, and there are many bulges with different sizes on the surface due to the escape of gas (Figure 3b). With the increase in temperatures, the powder completely loses the characteristics of raw materials and finally changes to a more brittle blocky structure, as shown in Figure 3c, which further reveals that the complex intermediates have formed due to the reaction of H_3_BO_3_ with CO(NH_2_)_2_.

The morphologies were characterized using SEM techniques, as observed in Figure 3d–f. Figure 3d shows that the precursor is composed of large blocks. Additionally, the surface is rough and no pores can be found, implying that there are no gases escaping at 85 °C. As seen in Figure 3e, the surface become relatively smooth and there are a small number of pores that exist, marked in red circles, indicating the presence of gas escape. When the treatment temperature is 250 °C, the surface become smoother (Figure 3f). This is because the decomposition products at 250 °C are mostly liquid phase, the smooth surface can be formed after the natural cooling and solidification. Meanwhile, a large number of pores are present, which is caused by the release of large amounts of CO_2_ and H_2_O, etc., gases derived from the decomposition of raw materials. The phenomenon is in agreement with the above FITR, XRD and photograph results, which suggests that the temperature has an effect on the morphology and structure of precursors and may further influence the final products.

### 2.2. Characterization of the h-BN Products

**Figure 4:**
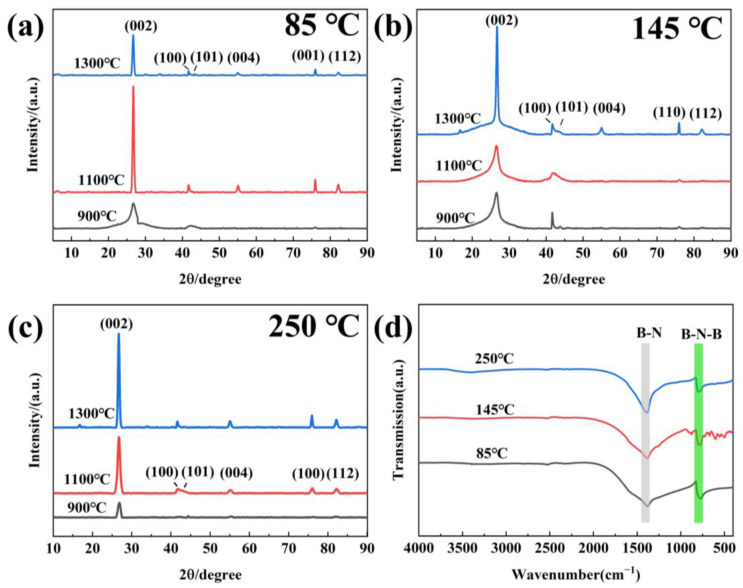
XRD (**a**–**c**) and FTIR (**d**) of h-BN products.

The precursors obtained under different temperatures were heated to 900, 1100 and 1300 °C for 3 h in N_2_ to investigate the effect on h-BN product. The XRD result is presented in Figure 4a–c. It can be observed that the diffraction peaks at 26°, 42°, 44°, 55°, 76° and 82° appear in all the samples, which can be indexed into the (002), (100), (101), (004), (110) and (112) facets of h-BN (JCPDS 34-421), respectively, indicating the successful synthesis of h-BN [27]. As for the sample obtained using 85 °C precursors in Figure 4a, when the sintering temperature (ST) increases from 900 °C to 1100 °C, the intensity of the h-BN characteristic peak gradually become strong, indicating that increase in the sintering temperature is conductive to improve the crystallization degree. With the temperature raising from 1100 °C to 1300 °C, the peak areas of (110) and (111) of h-BN increase and the GI index of h-BN decreases, which reveals that the three-dimensional ordering of the crystal improves, and the morphology grows towards a sheet-like structure [28,29]. For h-BN obtained using 145 and 250 °C precursors (Figure 4a,b), the peak intensity increases with the temperature increasing, revealing that the crystallization degree is greatly enhanced. Based on this, it can be deduced that the treatment temperature of the precursors has no effect on the product phase but has an impact on the crystallinity and ordering degree. The FTIR spectra of samples obtained at 1300 °C are shown in Figure 4d. Two strong peaks at 1420 cm^−1^ and 770 cm^−1^ can be clearly found and correspond to v(B–N) and δ(B–N–B) modes of h-BN, respectively [30,31]. Additionally, no other peaks can be observed, indicating that the h-BN is of high purity.

**Figure 5:**
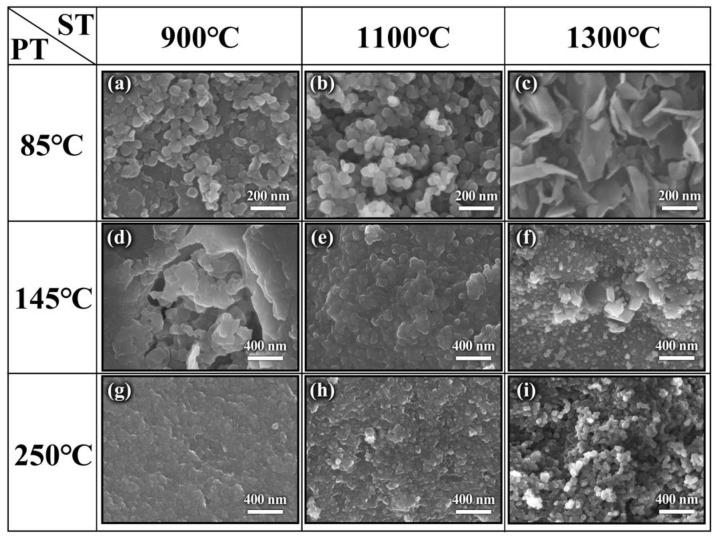
SEM images of h-BN under (**a**–**c**) PT—85 °C and ST—900, 1100 and 1300 °C; (**d**–**f**) PT—145 °C and ST—900, 1100 and 1300 °C; (**g**–**i**) PT—250 °C and ST—900, 1100 and 1300 °C.

**Figure 6:**
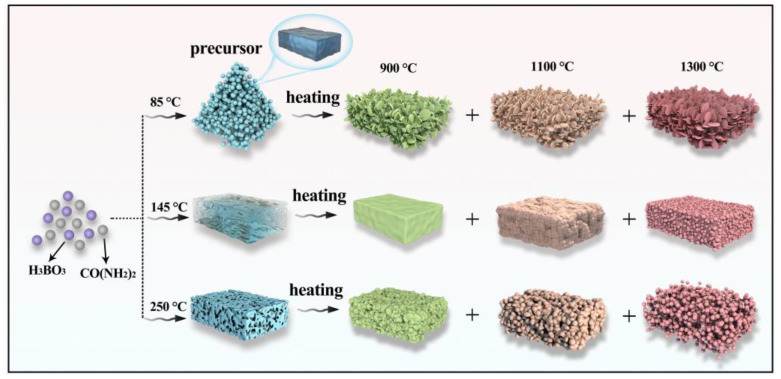
Schematic illustration of the formation process of h-BN with different morphology.

**Figure 7:**
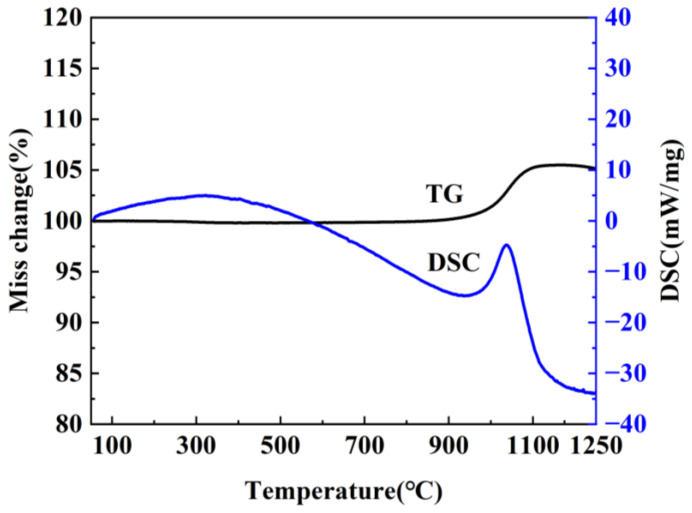
TG–DSC curve of the h-BN.

Figure 5 shows SEM images of the products. Compared with 85 °C precursors, the agglomerated h-BN nanoparticles with the average size of 70 nm have been formed and covered on the original block surface at 900 °C, as observed in Figure 5a. From Figure 5b, it can be seen that the particle size changes and is about 40–80 nm at 1100 °C, and the spherical structure becomes more uniform with the increase in temperature. Moreover, the dispersion is further improved. When the sintering temperature increases to 1300 °C, the spherical morphology disappears and transforms to flake structure with a diameter of 100 nm, which further confirms the XRD result of the GI index decrease. Figure 5a–c illustrate that h-BN crystallization degree and the lateral size of particles gradually increase as temperatures rise, which indicates that the morphology can be regulated by controlling the temperature. This is because NH_3_ that came from the precursor has a promoting effect on the growth of h-BN. As for the sample obtained using 145 °C precursors in Figure 5d–f, the product morphology at 900 °C (Figure 5d) is similar to its precursor, and the unobvious and cohesive spherical structure is beginning to form on the block surface. The cohesive spherical structure becomes more obvious and tends to separate from each other at 1100 °C, and the independently dispersed nanoparticles are obtained at 1300 °C, which can be explained by crystal growth kinetics [32,33]. The samples obtained using 250 °C precursors are shown in Figure 5g–i. As observed in Figure 5g, the nanoparticles have been formed on the surface of the bulk precursor. With the gradual increase in temperature, the spherical nanoparticles become more and more obvious, and the uniformity is gradually improved, and the size of h-BN nanospheres synthesized at 1300 °C is about 30–50 nm. Furthermore, the evolution of morphology and structure can be clearly observed, as shown in Figure 6. By comparing the XRD and SEM of different products, it can be found that the structure and morphology of the products display great differences under the same sintering temperature, and the spherical morphology and crystallinity will be enhanced with the sintering temperature increasing. In addition, the thermogravimetric analysis of the samples obtained at 1300 °C was performed at a heating rate of 5 °C min^−1^ under air atmosphere from room temperature to 1300 °C. As shown in Figure 7, it can be found that no mass change can be found until 1000 °C, indicating that the synthesized h-BN nanoparticles show high purity and good thermal stability.

Based on the above results, it can be concluded that both the treatment temperature of precursors and the sintering temperature of h-BN products have an influence on the morphology and structure. However, the influence of the precursor treatment temperature on the morphology of products is greater than the sintering temperature. The optimal conditions for obtaining high-quality spherical h-BN are the pretreatment temperature of 250 °C and sintering temperature of 1300 °C.

### 2.3. Formation Mechanism of h-BN

**Figure 8:**
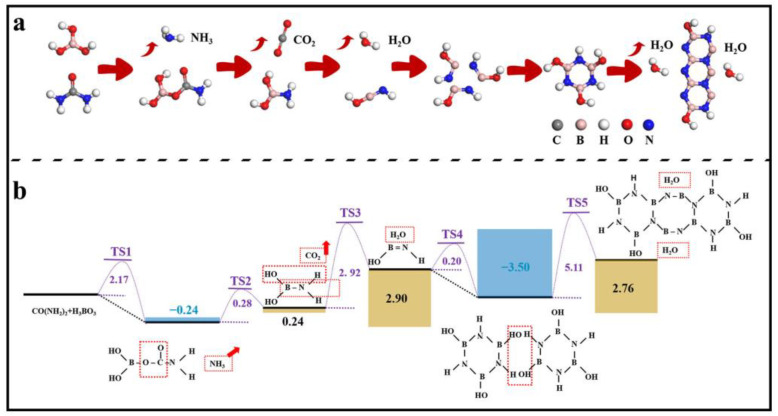
(**a**) Simulation of the formation process of h-BN and (**b**) the reaction pathway with energy barrier for each step.

To further explore the reaction process of CO(NH_2_)_2_ and H_3_BO_3_ to form h-BN, density functional theory (DFT) calculations were performed using Materials Studio’s DMol3 [34,35,36]. The reaction pathways were simulated based on frontier orbital theory, and the related structures are as shown in Figure 8. By searching for transition states (TSs) of each step, it can be found that the first H from H_3_BO_3_ transfers to –NH_2_ of CO(NH_2_)_2_ and combines with it to form NH_3_. Meanwhile, H4BCNO4 is also formed, and the corresponding energy barrier is 2.147 eV (TS 1). The breaking of the B–O bond and C–N bond in the reactants leads to the formation of B–N bond and CO_2_, and the barrier is only 0.28 eV (TS 2). As the temperature increases, the combination of –OH on B sites with H on N sites to form H_2_O and HO–B=N–H needs to overcome a barrier of 2.92 eV (TS 3). Subsequently, three HO–B=N–H molecules form a ring structure, and at this point, the energy of the products is 3.50 eV lower than that of the reactants, indicating that the ring structure formed by three HO–B=NH molecules is more stable, with a reaction barrier of 0.20 eV (TS 4). Finally, another two ring structures connect to –OH on B and H on N to form 2 H_2_O again and three ring structures. During the process, the H_2_O is easy to generate and leave the h-BN surface at high temperature, and the connection between B and N in the two ring structures also occurs. The related barrier is 5.11 eV (TS 5), which can be overcome at high reaction temperatures. Based on this, the ring structure continues to increase, and then h-BN is formed.

### 2.4. Specific Surface Area Analysis of h-BN

**Figure 9:**
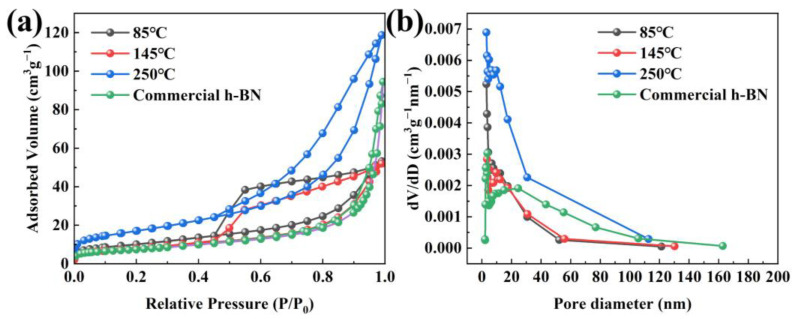
(**a**) Nitrogen adsorption–desorption isotherm and (**b**) corresponding pore size distributions of synthesized h-BN and commercial h-BN nanoparticles.

The specific surface area and pore diameter information of samples obtained at 1300 °C are achieved via the N_2_ adsorption–desorption isotherms, as shown in Figure 9. Figure 9a shows that the sample that came from 250 °C precursors possesses the highest specific surface area of 61.1 m^2^ g^−1^, which is caused by the formation of uniform and non-cohesive spherical nanoparticles using 250 °C precursors. In addition, the specific surface area (27.9 m^2^ g^−1^) of h-BN at 145 °C treatment temperature is lower than that of 85 °C (36.5 m^2^ g^−1^) due to nanoparticles anchored on the bulk matrix to form an entity at 145 °C. However, their specific surface area is higher than the commercial h-BN nanoparticles (26.2 m^2^ g^−1^), and their morphology is more uniform and the diameter is smaller, which can manifest in the sintering activity of the synthesized h-BN nanoparticles being correspondingly high [37,38]. Furthermore, the isotherm can be classified as type-IV isotherm with type H4 hysteresis loop at the range of 0.5–1.0 based on the IUPAC nomenclature [39], illustrating that there exist slit-shaped mesopores in the h-BN samples. The pore size distribution is calculated according to the BJH method, as seen in Figure 9b. It is worth noting that the mesoporous distribution of all samples has only a single peak, and most of the pore sizes are distributed between 3 and 12 nm, which indicates that the pore size distribution of all samples is relatively uniform [40,41]. Based on the BJH method, the average pore diameter of 85 °C, 145 °C and 250 °C is determined to be 3.07 nm, 4.29 nm and 3.06 nm, respectively, which is lower than that of the commercial h-BN nanoparticles (23.34 nm). The uniform and finer particles and the high specific area are beneficial to shorten the distance of diffusion and increase the solubility of particles in the liquid phase, which causes an acceleration of the sintering process and will lead to a preferable sintering activity.

## 3. Experimental Section

### 3.1. Preparation of h-BN Nanoparticles

**Figure 10:**
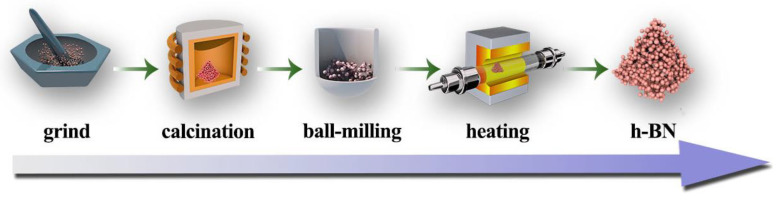
The preparation procedure of h-BN nanoparticles.

All the chemicals were of analytical grade (A.R.) and without further purification prior to use. The preparation procedure of h-BN nanoparticles includes two steps: (1) the formation of precursor via calcination and ball-milling treatment and (2) conversion of the precursor into h-BN nanoparticles in flowing N_2_, as illustrated in Figure 10. Firstly, 6 g of H_3_BO_3_ and 6.17 g of CO(NH_2_)_2_ were ground and mixed to form a homogeneous powder. Then, the powder was filled into a crucible and heated to a certain temperature, i.e., 85, 145 and 250 °C in air in a muffle furnace at the heating rate of 5 °C min^−1^ to obtain white powder. Subsequently, the powder was milled by conventional wet ball-milling method with ethanol as the medium at 200–300 rpm/min for 4 h to obtain uniform and fine precursor. Then, the obtained precursor was heated in a tube furnace at 900, 1100 and 1300 °C for 3 h in flowing N_2_. When the furnace was cooled to room temperature, the powder was soaked and rinsed with hot deionized water several times to remove the residual B_2_O_3_. Finally, the product was obtained by centrifugation and then drying in an oven.

### 3.2. Characterization

The phase and structure were examined by X-ray diffraction (XRD) on a PW1710 in diffractometer (Rigaku, Ultima IV, Osaka, Japan) with Cu Kα radiation over a 2*θ* ranging from 10 to 90° and Fourier transformation infrared spectroscopy (FTIR) using a Nicolet-Nexus 670 device (East Lyme, CT, USA) with spectral scanning between 4000 and 500 cm^−1^. The morphology and microstructure of the product were characterized using field emission scanning electron microscopy (FE-SEM) on a JSM-6701F (JEOL, Akishima, Japan). The thermogravimetric analysis was carried out on a Netzsch STA 449C thermal analyzer (Selb, Germany). The specific surface area was determined from the nitrogen adsorption–desorption isotherm measured at 77 K on a Quadrasorb SI-MP analyzer (Boynton Beach, FL, USA) using the Brunauer–Emmett–Teller (BET) model.

## 4. Conclusions

The BN nanoparticles with different morphologies were synthesized by different precursors obtained at the required temperature (i.e., 85, 145 and 250 °C) using H_3_BO_3_ and CO(NH_2_)_2_ as raw materials. Multiple techniques including XRD, FTIR, SEM and BET were used to characterize the nanoparticles. Based on the characterization and DFT calculations, the conclusions are summarized as follows.

(1) The morphology and structure of h-BN products are significantly affected by the treatment temperature of precursors. When the sintering temperature is 1300 °C, the morphology gradually transforms from flake-like to sphere structure with the increase in treatment temperature.
(2) The crystallinity and dispersibility will be greatly improved with the sintering temperature increasing. The optimal condition for obtaining high-quality spherical h-BN with size of 30–50 nm is the pretreatment temperature of 250 °C and sintering temperature of 1300 °C.
(3) The h-BN obtained using 250 °C precursors shows a higher specific surface area (61.1 m^2^ g^−1^) than that of 145 °C (27.9 m^2^ g^−1^) and 85 °C (36.5 m^2^ g^−1^), indicating that it will present higher sintering activity.
(4) The reaction pathway and formation mechanism of h-BN have been revealed by DFT calculations. TS search shows that the five stationary states and five transition states exist in the reaction pathway. Additionally, boric acid and urea are easily combined to form intermediates, and the calculated barrier can be overcome at high temperatures to form a ring h-BN structure.

## Data Availability

Data are contained within the article.
